# Supplementary material for: MetaRibo-Seq measures translation in microbiomes
Source: Nat Commun. 2020 Jun 29;11:3268. doi: 10.1038/s41467-020-17081-z (PMC7324362; doi:10.1038/s41467-020-17081-z)
Supplement: Supplementary file 10 — Supplementary Data 7 [file 41467_2020_17081_MOESM10_ESM.zip › File2/Confidence_High_Taxonomy/163522_out.krona.html]

Javascript must be enabled to view this page.

members
magnitude
magnitudeUnassigned
count
unassigned
taxon
rank

163522\_out

3

2
superkingdom
3

3
1239
phylum

186801
class
3

186802
order
3

3
990719
family

1930012
species
3

SRS020869\_contig\_number\_contig-100\_1709.202340SRS1041140\_contig\_number\_18875SRS148196\_contig\_number\_5564
